# Correlations between Endomyocardial Biopsies and Cardiac Manifestations in Taiwanese Patients with the Chinese Hotspot IVS4+919G>A Mutation: Data from the Fabry Outcome Survey

**DOI:** 10.3390/ijms18010119

**Published:** 2017-01-09

**Authors:** Ting-Rong Hsu, Fu-Pang Chang, Tzu-Hung Chu, Shih-Hsien Sung, Svetlana Bizjajeva, Wen-Chung Yu, Dau-Ming Niu

**Affiliations:** 1 Department of Pediatrics, Taipei Veterans General Hospital, Taipei 112, Taiwan; romberg@gmail.com (T.-R.H.); mno4.chu@gmail.com (T.-H.C.); 2 Institute of Clinical Medicine, National Yang-Ming University, Taipei 112, Taiwan; orimiltea@gmail.com; 3 Department of Pathology and Laboratory Medicine, Taipei Veterans General Hospital, Taipei 112, Taiwan; 4 Division of Cardiology, Department of Medicine, Taipei Veterans General Hospital and National Yang-Ming University, Taipei 112, Taiwan; shsang@vghtpe.gov.tw; 5 Shire, 6300 Zug, Switzerland; sbizjajeva@shire.com

**Keywords:** Fabry disease, Fabry Outcome Survey, endomyocardial biopsy, enzyme replacement therapy, hypertrophic cardiomyopathy

## Abstract

We retrospectively evaluated correlations between cardiac manifestations and globotriaosylceramide (Gb3) accumulation in cardiomyocytes from Taiwanese patients with Fabry disease and the IVS4+919G>A (IVS4) mutation who underwent endomyocardial biopsy (Shire; Fabry Outcome Survey data; extracted January 2015). Of 24 males and six females (median age [Q1; Q3] at biopsy 60.4 [57.4; 64.1] and 61.3 [60.4; 65.1] years, respectively), 13 males (54.2%) and five females (83.3%) received agalsidase alfa enzyme replacement therapy (ERT) before biopsy. Median left ventricular mass indexed to height (LVMI) within ±6 months of biopsy was 65.3 (52.7; 93.1) in males and 53.2 (42.0; 55.0) g/m^2.7^ in females. A moderate, positive, statistically significant correlation was found between the percentage area Gb3 accumulation in cardiomyocytes and LVMI (Spearman’s ρ, 0.45; *p* = 0.014); a smaller, positive, non-statistically significant correlation was observed between cardiomyocyte diameter and LVMI (Spearman’s ρ 0.16, *p* = 0.394). Moderate, statistically significant, negative correlations were found between Gb3 accumulation and ERT duration (Spearman’s ρ, −0.49, *p* = 0.007) and between cardiomyocyte size and ERT duration (Spearman’s ρ, −0.37, *p* = 0.048). Longer ERT duration was associated with smaller amounts of Gb3 accumulation and smaller cardiomyocyte size. Further follow-up is recommended to confirm these trends in a larger sample size.

## 1. Introduction

Progressive accumulation of glycosphingolipids, mainly globotriaosylceramide (Gb3), in cellular lysosomes throughout the body, resulting from a genetic deficiency in α-galactosidase A activity, leads to the wide variety of signs and symptoms typically found in Fabry disease. Clinical features of classical Fabry disease tend to first appear during childhood and adolescence (e.g., acroparesthesia, pain, angiokeratomas) and progress throughout adulthood to end-stage organ disease, peaking in the fifth decade for males and the seventh for females [1].

Cardiac [2,3], renal [4], and cerebrovascular [5] variants of Fabry disease have been reported. In patients with the cardiac variant, disease manifestations generally occur later in life than in patients with classical Fabry disease, and are mainly restricted to the heart [2,3]. More recent reports have provided evidence indicating that these variants could actually be more common than originally thought. For example, the later-onset cardiac IVS4+919G>A (IVS4) mutation was found at a high frequency in the Taiwanese population among both newborns [6,7] and patients diagnosed with idiopathic hypertrophic cardiomyopathy [6]. However, the relationships between pathological characteristics and cardiac manifestations in patients with this later-onset variant of Fabry disease are not yet well understood.

Enzyme replacement therapy (ERT) has shown good potential in slowing the progression of, and perhaps even reversing, Fabry disease [8,9,10]. In Taiwan, to qualify for reimbursement for ERT, the Bureau of National Health Insurance stipulates that patients must undergo endomyocardial biopsy to confirm that Fabry disease is the main cause of their hypertrophic cardiomyopathy. This requirement provides an ideal opportunity for a closer examination of cardiac pathological changes in Taiwanese patients with the later-onset IVS4 mutation [11].

The objective of this analysis was to evaluate endomyocardial biopsy results and cardiac manifestations in Taiwanese patients with the IVS4 mutation who are registered in the Fabry Outcome Survey (FOS).

## 2. Results

As of January 2015, data were available from the FOS for 30 Taiwanese patients (24 males and six females) who had the IVS4 mutation and who also underwent endomyocardial biopsy at any point. Before biopsy, five females (83.3%) and 13 males (54.2%) received ERT with agalsidase alfa.

Males and females were comparable in terms of age at symptom onset, age at diagnosis, and age at start of treatment (Table 1). Similarly, the median age (IQR) at biopsy was 60.4 (57.4; 64.1) years in males and 61.3 (60.4; 65.1) years in females (Table 1). One female (16.7%) and 10 males (41.7%) underwent biopsy before ERT was initiated.

Comparing cardiac parameters within ±6 months of the biopsy date, we found a greater median (IQR) left ventricular mass indexed to height (LVMI) of 65.3 (52.7; 93.1) g/m^2.7^ in males compared with 53.2 (42.0; 55.0) g/m^2.7^ in females (Table 1; one male did not have LVMI data within ±6 months of the biopsy date and was excluded from this analysis). Median ventricular wall thickness was also greater in males than females (14.5 [12.0; 18.0] vs. 10.8 [10.0; 13.5] mm), as was the median diastolic blood pressure (BP) (76.0 [72.5; 82.0] vs. 71.0 [64.0; 73.0] mmHg; Table 1). Systolic BP and midwall fractional shortening were similar in both sexes (Table 1).

Regarding renal parameters within ±6 months of the biopsy date, the median (IQR) estimated glomerular filtration rate (eGFR) using the Modification of Diet in Renal Disease (MDRD) was slightly greater in males than females (79.8 [66.1; 92.1] vs. 73.2 [55.0; 86.5] mL/min/1.73 m^2^), whereas the creatinine level was similar in both sexes (Table 1).

The median (IQR) area of Gb3 accumulation in cardiomyocytes was similar in males and females (0.10% [0.06%; 0.12%] vs. 0.05% [0.03%; 0.11%]). Furthermore, no difference was observed in the median cardiomyocyte size between males and females (24.1 [23.2; 26.1] vs. 24.1 [23.5; 25.8] µm).

**Figure 1:**
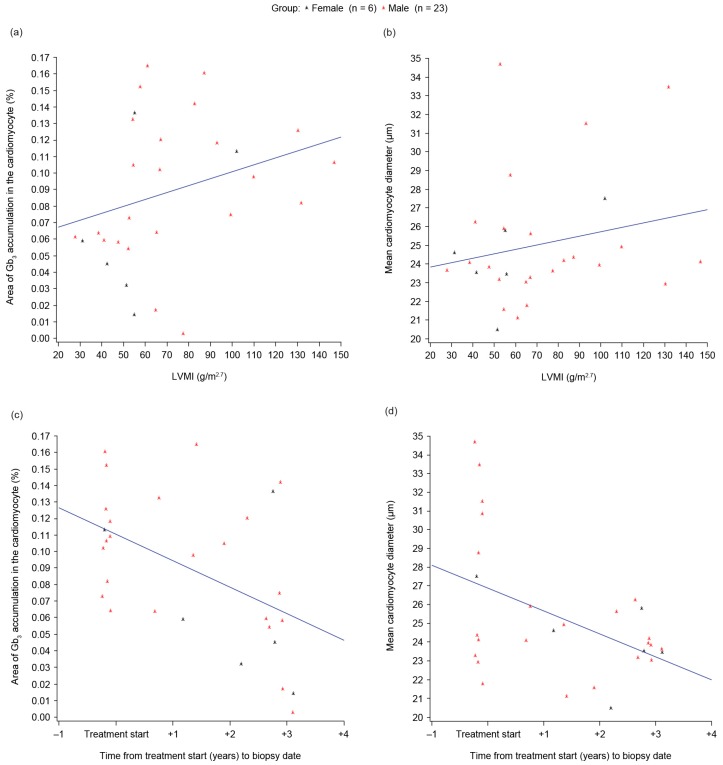
Scatter plots illustrating: (**a**) Globotriaosylceramide (Gb3) accumulation by left ventricular mass indexed to height (LVMI); (**b**) Cardiomyocyte size by LVMI; (**c**) Gb3 accumulation by time from treatment start to biopsy date; (**d**) Cardiomyocyte size by time from treatment start to biopsy date. Left ventricular hypertrophy was defined as LVMI of >51 g/m^2.7^ in males and >48 g/m^2.7^ in females [12].

A positive correlation was found between the percentage area of Gb3 accumulation within cardiomyocytes and LVMI in our overall IVS4 population (Spearman’s correlation coefficient 0.45, *p* = 0.014; Figure 1a). A positive correlation of a smaller size was observed between the cardiomyocyte size and LVMI (Spearman’s correlation coefficient 0.16, *p* = 0.394; Figure 1b), although this correlation was not statistically significant.

A negative correlation was found between Gb3 accumulation and the duration of ERT (Spearman’s correlation coefficient −0.49, *p* = 0.007; Figure 1c) and also between the cardiomyocyte size and the duration of ERT (Spearman’s correlation coefficient −0.37, *p* = 0.048; Figure 1d), both of which were statistically significant.

## 3. Discussion

This is the first study comparing endomyocardial biopsy results and cardiac parameters in Fabry disease. We found a statistically significant positive correlation between the area of Gb3 accumulation within cardiomyocytes and LVMI; a similar positive correlation was also observed between cardiomyocyte size and LVMI, although this was not statistically significant. We also found negative correlations between cardiomyocyte size and the time from the start of treatment, and between Gb3 accumulation and the time from the start of treatment, both of which were statistically significant. The latter observation is in agreement with results from our previous analysis, in which a relatively small amount of Gb3 accumulation was observed in the cardiomyocytes of patients who had received ERT for the longest duration [11]. Despite reports of limited Gb3 clearance from cardiomyocytes in patients receiving ERT [13,14], our findings could provide further evidence to suggest that ERT may be beneficial in patients with later-onset Fabry disease.

Histological findings of Gb3 deposition in the cardiomyocytes can be an early indicator of cardiac involvement before signs and symptoms manifest, particularly left ventricular hypertrophy [11,15]. Earlier diagnosis and treatment initiation are expected to have beneficial consequences for the management of classical Fabry disease, and also for the later-onset cardiac variant [16].

Regarding the mechanism of hypertrophic cardiomyopathy in Fabry disease, it has been hypothesized that the accumulation of Gb3 triggers a number of pathophysiological processes, such as inflammatory reactions, which, when combined with the activity of growth-promoting factors such as plasma globotriaosylsphingosine and sphingosine-1-phosphate, or other genetic or environmental factors, may result in progressive cardiovascular remodeling [17,18]. Thus, it is likely that Gb3 accumulation is the main trigger of cardiac hypertrophy, but not its main cause. Consistent with this hypothesis, in our study, the percentage area of Gb3 accumulation was only around 0.1%–0.2% of the area of the cardiomyocytes.

This analysis is retrospective and uses data from the FOS registry, which was not specifically designed to collect data on the parameters investigated herein; furthermore, the inclusion criteria are less rigid than would be required for a clinical trial. The small sample size, particularly for females, is a further limiting factor and means that the statistical analysis must be considered exploratory and descriptive.

We have previously reported results from endomyocardial biopsies in Taiwanese patients that strongly supported the role of IVS4 as a pathogenic later-onset Fabry mutation [11], and the results herein provide further evidence to support this. Further follow-up analyses and the collection of additional data are required to confirm the trends reported here and to increase the knowledge base of cardiac pathology in later-onset IVS4 Fabry disease.

## 4. Materials and Methods

### 4.1. Patients and Study Design

This was a retrospective analysis of data entered in FOS, an international outcomes database sponsored by Shire for the collection of data on the natural course and treatment of Fabry disease. Patients with confirmed Fabry disease who are receiving, or are candidates for, ERT with agalsidase alfa are eligible for inclusion in FOS. All procedures followed are in accordance with the ethical standards of the responsible committees on human experimentation (institutional and national) and with the Helsinki Declaration of 1975, as revised in 2000. The participation of each center in FOS has been approved by the respective ethics committees or institutional review boards. All patients gave written, informed consent before their data were entered into FOS.

The inclusion criteria for this analysis were Taiwanese patients who carried the Chinese hotspot IVS4+919G>A mutation, as confirmed by molecular analysis, who also underwent endomyocardial biopsy at any point.

The FOS data used for this analysis were extracted from the FOS database in January 2015.

### 4.2. Clinical Assessments

Data from all routine clinical assessments undertaken at each visit are entered into the FOS database but, for the purposes of this analysis, only values closest to the biopsy date (within ±6 months) for the following assessments were included: diastolic and systolic BP, eGFR using the MDRD equation, and serum creatinine.

Echocardiography to determine left ventricular mass indexed to height (LVMI g/m^2.7^) was performed according to prespecified FOS guidelines and using recommendations published by the American Society of Echocardiography [19]. Additional echocardiographic measurements included midwall fractional shortening and ventricular wall thickness. Only values closest to the biopsy date (within ±6 months) were analyzed.

### 4.3. Cardiac Pathology

Endomyocardial biopsy using a flexible endomyocardial bioptome was performed to obtain endomyocardial tissue from the right side of the interventricular septum. Then, cardiomyocyte size and percentage area of Gb3 accumulation were assessed in cardiomyocytes from these endomyocardial tissues.

Cardiomyocyte diameter was obtained from transverse sections by measuring the minor axis at the nuclear level, as performed in our previous study [11]. A total of 15 cells from three micrographs were measured for each sample.

The average percentage area of Gb3 accumulation in cardiomyocytes on toluidine blue stained micrographs was obtained using ImageJ software, an image processing program developed by the US National Institutes of Health (Bethesda, MD, USA) (available for download at http://rsb.info.nih.gov/ij/).

**Figure 2:**
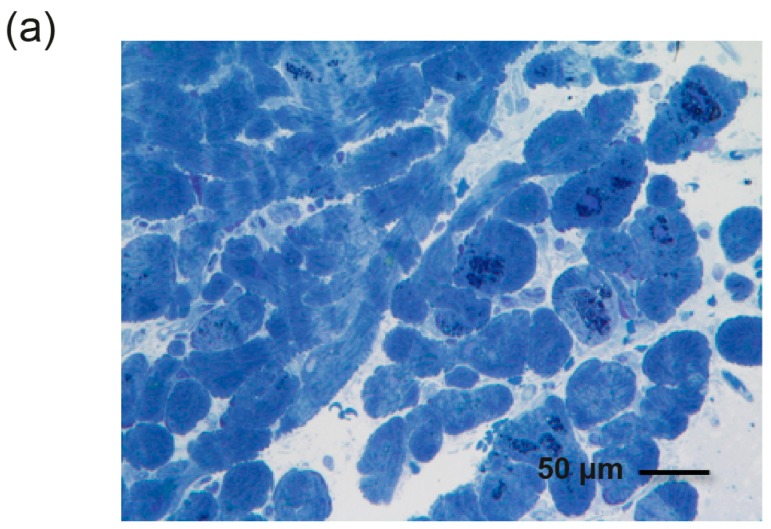

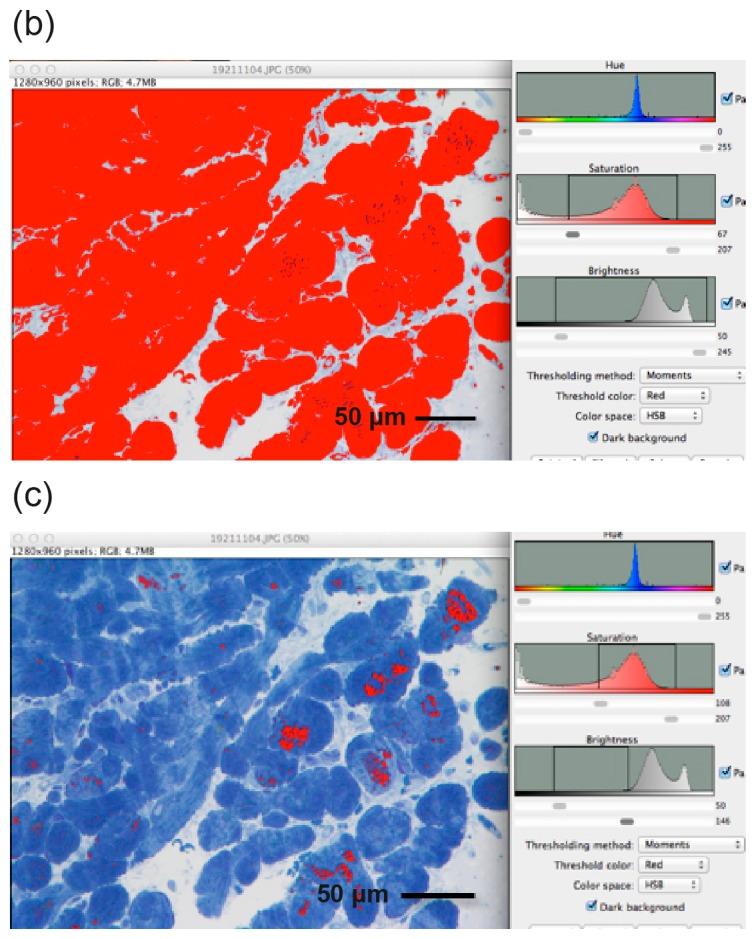
Image processing using ImageJ software. (**a**) Cardiomyocyte sample from a single Fabry patient with toluidine blue staining; (**b**) Total area of the cardiomyocytes (in red); (**c**) Area of globotriaosylceramide (Gb3) deposition within the cardiomyocytes. The percentage area of Gb3 deposition was calculated from these two values, using ImageJ.

The total area of cardiomyocytes within a micrograph (Figure 2a) was obtained by adjusting the color threshold tool to select and measure the desired area (Figure 2b). The color threshold tool was then used to select areas of toluidine blue staining to measure the area of Gb3 accumulation within the cardiomyocytes (Figure 2c). The percentage of Gb3 deposition was calculated within ImageJ using these two values. All histopathological specimens were reviewed by the same pathologist, who was blinded to the clinical information.

### 4.4. Statistical Analysis

Due to the small sample size, non-parametric measures were used. Descriptive statistics were calculated for the demographic and clinical characteristics. Spearman’s rank correlation coefficient was calculated to assess the relationship between cardiac parameters and cardiac biopsy results. Statistical significance was set at 5%, and SAS software Version 9.2 (SAS Institute Inc., Cary, NC, USA) was used.

## 5. Conclusions

The correlation between LVMI and Gb3 accumulation in cardiomyocytes was moderate but statistically significant in our IVS4 patients. Furthermore, in the patients treated with ERT, both Gb3 accumulation and cardiomyocyte size showed moderate, statistically significant, negative correlations with time from the start of treatment to the biopsy date; thus, longer treatment periods were associated with smaller amounts of Gb3 accumulation and smaller cardiomyocyte size.

## Figures and Tables

**Table 1:** Demographic characteristics and cardiac and renal parameters within ±6 months of the biopsy date.

Characteristics and Parameters, Median (Q1; Q3) Years	Male (*n* = 24)	Female (*n* = 6)
**Demographic Characteristics**		
Age at symptom onset	49.0 (47.0; 54.0); *n* = 14	48.0 (47.0; 48.0); *n* = 5
Age at diagnosis	59.0 (53.5; 63.0)	59.5 (57.0; 62.0)
Age at FOS entry	60.1 (56.9; 64.2)	61.1 (60.1; 64.4)
Age at treatment start	59.9 (54.6; 64.2); *n* = 23	59.9 (58.9; 62.9)
Age at biopsy	60.4 (57.4; 64.1)	61.3 (60.4; 65.1)
**Cardiac Parameters**		
LVMI, g/m^2.7^	65.3 (52.7; 93.1); *n* = 23	53.2 (42.0; 55.0)
Midwall fractional shortening, %	14.6 (11.5; 16.7); *n* = 20	15.1 (12.3; 18.3)
Diastolic BP, mmHg	76.0 (72.5; 82.0)	71.0 (64.0; 73.0); *n* = 5
Systolic BP, mmHg	120.0 (115.5; 129.0)	119.0 (106.0; 121.0); *n* = 5
MVWT, mm	14.5 (12.0; 18.0); *n* = 23	10.8 (10.0; 13.5)
**Renal Parameters**		
eGFR MDRD, mL/min/1.73 m^2^	79.8 (66.1; 92.1)	73.2 (55.0; 86.5)
Creatinine, mg/dL	1.0 (0.9; 1.2)	0.8 (0.7; 1.1)

BP, blood pressure; eGFR, estimated glomerular filtration rate; FOS, Fabry Outcome Survey; LVMI, left ventricular mass indexed to height; MDRD, Modification of Diet in Renal Disease; MVWT, mean ventricular wall thickness.
